# Energy Therapies in Advanced Practice Oncology: An Evidence-Informed Practice Approach

**Published:** 2013-05-01

**Authors:** Pamela J. Potter

**Affiliations:** From University of Portland School of Nursing, Portland, Oregon

## Abstract

Advanced practitioners in oncology want patients to receive state-of-the-art care and support for their healing process. Evidence-informed practice (EIP), an approach to evaluating evidence for clinical practice, considers the varieties of evidence in the context of patient preference and condition as well as practitioner knowledge and experience. This article offers an EIP approach to energy therapies, namely, Therapeutic Touch (TT), Healing Touch (HT), and *Reiki*, as supportive interventions in cancer care; a description of the author’s professional experience with TT, HT, and *Reiki* in practice and research; an overview of the three energy healing modalities; a review of nine clinical studies related to oncology; and recommendations for EIP. These studies demonstrate a response to previous research design critiques. Findings indicate a positive benefit for oncology patients in the realms of pain, quality of life, fatigue, health function, and mood. Directionality of healing in immune response and cell line studies affirms the usual explanation that these therapies bring harmony and balance to the system in the direction of health. Foremost, the research literature demonstrates the safety of these therapies. In order to consider the varieties of evidence for TT, HT, and *Reiki*, EIP requires a qualitative examination of patient experiences with these modalities, exploration of where these modalities have been integrated into cancer care and how the practice works in the oncology setting, and discovery of the impact of implementation on provider practice and self-care. Next steps toward EIP require fleshing out the experience of these modalities by patients and health-care providers in the oncology care setting.

For advanced practice professionals in the oncology setting, state-of-the-art care and support for patients during the healing process are of paramount importance. An analysis of National Health Statistics data found that approximately 30% of people with lung, breast, colon, or prostate cancer use complementary therapies (Fouladbakhsh & Stommel, 2010). We are seeing an increase in the implementation of noninvasive supportive therapies in oncology settings, including Therapeutic Touch (TT), Healing Touch (HT), and *Reiki* (Pierce, 2007).

Because of this increase, practitioners are justly concerned about the safety and efficacy of the nonconventional therapies that patients might choose. Historically, a great divide has separated the conventional from the unproven. As an approach to care, integrative medicine provides a bridge for considering these therapies in the cancer setting (Bravewell Collaborative, 2011). Centered on the patient, integrative medicine addresses the varied influences affecting health, personalizes care, and chooses the most appropriate treatments to facilitate health and healing. Integrative oncology, within the greater definition of integrative medicine, chooses therapies to enhance medical treatment efficacy and to "improve symptom control, alleviate patient distress and reduce suffering" (Sagar, 2006, p. 27). Likewise, integrative nursing, *informed by evidence*, "utilizes the full range of therapeutic modalities to support/augment the healing process, from least to most invasive" (M. J. Kreitzer, personal communication, October 25, 2012).

Evidence-informed practice (EIP), an approach to evaluating evidence for clinical practice, has been defined as "the integration of patient presentation and preferences, clinical experience and research in healthcare delivery" (Northwestern Health Sciences University, 2012a). Evidence-informed practice, a term drawn from social sciences (Nevo & Slonim-Nevo, 2011) and increasingly used by integrative health/medicine practitioners, takes a different perspective from the evidence-based practice hierarchy of evidence that focuses on outcomes of randomized controlled trials (RCTs) while diminishing other forms of evidence. Evidence-informed practice broadens the understanding of evidence to include qualitative as well as quantitative research findings. Laboratory studies, RCTs, and meta-analyses provide explanations for "causal attributions and mechanisms of action" or "links between specific interventions with specific outcomes," whereas qualitative case reports, epidemiologic outcomes, and health services research provide "information about relevance and utility of practice, both proven and unproven" (Jonas, 2005, p. 80).

Evidence-informed practice includes the modality’s meaning for patients, its applicability to clinical practice, and its impact on greater clinical outcomes (especially costs). Evidence-informed practice establishes equipoise among clinical expertise, research evidence, and patient preference. As articulated by the Northwestern Health Sciences University (2012b), EIP is "where the best available science works in harmony with clinical expertise and patient preferences."

This article offers an EIP approach to energy therapies (TT, HT, *Reiki*) as supportive interventions in cancer care. Evidence-informed practice considers the varieties of evidence in the context of patient preferences and conditions in addition to practitioner knowledge and experience. This article provides the author’s professional experience with these modalities in practice and research, describes the three energy healing modalities, reviews quantitative studies related to oncology and discussion of the evidence, and makes recommendations for EIP.

Practitioners in oncology are concerned with alleviating suffering for their patients, and they are interested in interventions that do that and do no harm. At the same time, they want evidence to support the use of such therapies. This is best practice.

## Energy Therapies

Therapeutic Touch, Healing Touch, and *Reiki* are common biofield therapies offered by providers in the health-care setting. Practitioners in oncology have described evidence for a positive impact of biofield therapies with people experiencing symptoms associated with cancer diagnosis, treatment, and recovery (Coakley & Barron, 2012). The National Center for Complementary/Alternative Medicine (NCCAM, 2000) has described biofield therapies as those energy therapies that manipulate energy fields that theoretically surround and penetrate the body. Pierce (2007) lists common assumptions shared by biofield therapy practitioners of therapies like TT, HT, and *Reiki* (pp. 253–254):

- The human body has a subtle energy system that interpenetrates the physical anatomy and extends outward beyond it.
- The subtle energy may be conceptualized as universal energy or vital energy flowing through and available to all beings.
- The normal self-healing capacity of the human body is supported by the free and balanced flow of energy through its subtle energy system.
- Disease or disorder can be detected in the energy system (perhaps before it manifests in the physical body) and can be affected therapeutically by the action of energy practitioners, in support of the self-healing capacity of the body.
- Conscious healing intent and compassion are considered essential to the effectiveness of biofield therapies.
- Practitioners’ hands may or may not touch the body. Practitioners also may carry out healing work mentally, from a distance.

## THERAPEUTIC TOUCH

The Therapeutic Touch International Association (TTIA, 2012) describes TT as "a contemporary interpretation of several ancient healing practices…an intentionally directed process of energy exchange during which the practitioner uses the hands as a focus to facilitate the rebalancing of another’s energy field in support of healing." 

Therapeutic Touch facilitates relaxation and feelings of well-being. It restores balance through mobilizing the person’s own healing energies for restoring that balance. This is understood to facilitate the body’s innate healing processes, thereby relieving stress; supporting immune function, wound healing, and bone repair; and decreasing side effects of cancer treatment. Treatment response is individualized; repeated treatments may be required. Therapeutic Touch can also be used for maintaining balance in the healthy individual (TTIA, 2012).

The history of TT is one of observation, application, and inquiry. In the early 1970s, Dolores Krieger, a nursing professor affiliated with New York University, in collaboration with Dora Kunz, an intuitive healer and president of the Theosophical Society, observed the "laying on of hands" healing practices of Oskar Estebany, a renowned healer (Straneva, 2000). Reasoning that healing is something that could be learned, Krieger established a mentoring relationship with Kunz. Therapeutic Touch evolved from their observation and experience of many healing encounters into a systematic process of assessing, mobilizing, and directing energy toward healing. Forty years later, approximately 100,000 people have been trained in TT throughout the world (TTIA, 2012).

Although anyone can learn the basics of TT, it is a discipline requiring practice. The TTIA (2012) currently offers credentialing as a qualified Therapeutic Touch practitioner (QTTP) and a qualified Therapeutic Touch teacher (QTTT). Practitioner certification requires completion of basic and intermediate TT programs with a qualified teacher, a 1-year mentorship with a qualified practitioner/teacher, and case study documentation of single and longitudinal TT sessions.

Generally a noncontact intervention, TT is an individualized therapy administered in the recipient’s energy field through the healer’s hands. During treatment, which lasts for 10 to 20 minutes, the recipient (fully clothed) sits in a chair or lies on a massage table. A five-step process, which parallels the basic nursing process, includes (1) centering in the present moment, (2) assessing the energy field by holding the hands 2 to 6 inches from the body while moving them methodically downward from head to foot, (3) intervention to mobilize the energy field, (4) balancing/rebalancing through directing energy toward healing, and (5) evaluation/closure, which includes a final assessment (TTIA, 2012).

## HEALING TOUCH

Healing Touch is described as a relaxing, nurturing energy therapy administered through gentle touch to balance mental, emotional, spiritual, and physical well-being, thus supporting the person’s natural ability to heal (Healing Touch International [HTI], 2012). As described by HTI, "Healing Touch is a biofield therapy that encompasses a group of non-invasive techniques that utilize the hands to clear, energize, and balance the human and environmental energy fields" (HTI, 2012). Practitioners of HT use their hands in a heart-centered and intentional way to influence the human energy system (the energy field around the body and energy centers called *chakras* [defined below]); the caring relationship energetically facilitates health and healing (Healing Touch Program [HTP], 2012).

In the early 1980s, Janet Mentgen, a nurse in clinical practice, began to study complementary therapies—in particular, energy therapies like TT. In the nursing tradition, she began to teach what she had learned. As her teaching evolved, Mentgen included techniques from other healers and ones she intuitively developed, e.g., the chakra connection, mind clearance, and chakra spread, among others. The Colorado Center for Healing Touch was formed as the teaching organization for this program (HTP, 2012).

Currently, two organizations offer HT classes and certification: the Healing Touch Program and Healing Touch International (HTP, 2012; HTI, 2012). The HTP has six levels of instruction, while HTI has five. These courses guide the student through introductory energy therapy concepts, then move to more complex assessment and intervention, and conclude with higher-level interventions and instructions for establishing practice. At the highest level, through observation and demonstration, certified practitioners learn how to teach the lower-level courses.

During HT treatment, which generally lasts 40 to 60 minutes, the recipient lies fully clothed on a massage table. Similar to TT, the practitioner follows the basic steps of assessment, treatment/balancing, and evaluation. With hands several inches off the body, assessment involves observation for imbalances presenting as differences in temperature or sensation in any area of the person’s energy field, including the *chakras*. Originating in the Vedic or East Indian tradition, chakra is a Sanskrit word meaning "wheel." Seven *chakras*, located at strategic regions on the front and back of the body (root, sacral, solar plexus, heart, throat, brow, and crown), act as transducers for the energetic body. Treatment includes both gentle touch and nontouch techniques over various body areas to balance the person’s energy field, including the *chakras*. Brief treatments can also be offered when circumstances limit time and access. Recipients report relaxation, pain relief, decreased anxiety, and an increased sense of well-being (HTP, 2012; HTI, 2012).

## REIKI

*Reiki*, described as "spiritually directed life energy" (Rand, 1991, p. I-3), is a gentle hands-on spiritual healing tradition (Barnett & Chambers, 1996) originating from Japan. The word *Reiki*, meaning universal life energy, denotes an ancient system of healing that was rediscovered in the late 1800s by Mikao Usui, a Japanese Buddhist monk. Hawayo Takata, a Japanese-Hawaiian woman who greatly benefited from this modality, began teaching it in Hawaii in the mid-1930s, taking it to the US mainland in the early 1970s. In the tradition of the Japanese sensei (teacher), Reiki is passed on from master to student through attunement, an initiatory ceremony facilitated through the laying on of hands. This attunement is understood to open the student’s energy channels, thus facilitating the flow of universal life energy for treating others and oneself. Reiki master teachers trace their lineage back to Usui.

Generally, Reiki is taught at three levels: basic hands-on healing, distance healing, and master teacher. Although students learn basic hand placements, the "teaching" in Reiki lies in the practice. By offering Reiki, the practitioner receives benefit from the same universal life energy that flows to the recipient. The more the practitioner treats self and others, the more in tune he or she is with energy flow and balance. Each course level with its corresponding attunement raises the practitioner’s vibrations to higher healing frequencies. The master level prepares practitioners as teachers who can then pass on attunements to students.

Over the years, Reiki classes have evolved with much iteration. Some Reiki masters advocate a traditional approach to learning Reiki through the three levels and a strict apprenticeship over several years. Other masters offer the first two levels at the same time. Although the idea of *chakras* was not initially taught by Usui and his lineage, some teachers have since added these concepts to the courses they teach. While no official certifying organization offers credentialing for Reiki, courses in Reiki (as well as TT and HT) often include continuing education units from recognized provider organizations.

Reiki is understood to be present in every healing encounter: "hands on, Reiki on." Reiki may be offered as a whole treatment lasting 30 to 90 minutes, with a person lying on a massage table or seated in a chair. Reiki may also be offered briefly for comfort. There is no assessment or attempt to manipulate or balance the energies. The Reiki method allows the flow of universal life energy to the recipient, who in turn uses it where needed. Recipients experience deep relaxation, relief from anxiety and pain, and an increased sense of well-being. Practitioners report similar results (Potter, 2003).

## Biofield Therapy Efficacy

Originating in the crucible of nursing, TT has undergone the most extensive research of the three biofield therapies: 40 years of learning how to ask questions about the efficacy of this biofield therapy. Also with a foundation in nursing, HT, while newer on the research scene, demonstrates learning from TT research and its own research attempts to validate biofield healing. Reiki, practiced by nurses, health-care professionals, and lay healers, is newest to biofield efficacy research. What follows is a review of the research on these modalities, with an emphasis on implications for cancer care (see Table 1).

**Table 1:**
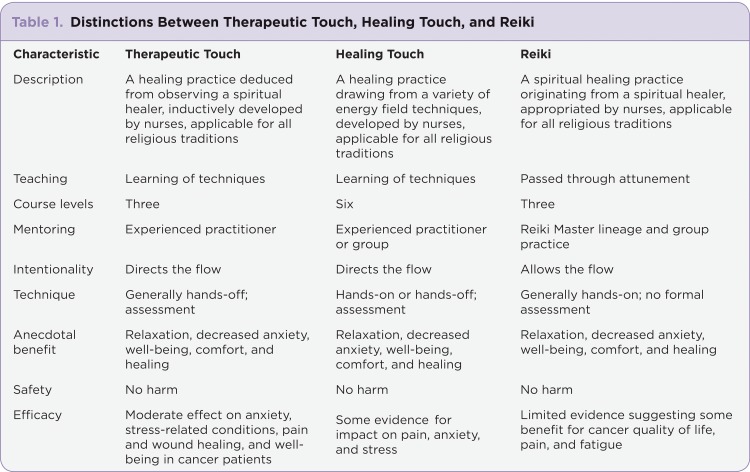
Table 1. Distinctions Between Therapeutic Touch, Healing Touch, and Reiki

## THERAPEUTIC TOUCH RESEARCH

Reviews of early TT research based on average effect size found a moderate effect on anxiety, stress-related conditions, pain, and wound healing (Peters, 1999; Winstead-Fry & Kijek, 1999). A longitudinal review of pain studies demonstrated significant decrease in chronic pain (Monroe, 2009). Natural Standard (2012a) assigned B grades—good scientific evidence—for the use of TT for anxiety, pain, stress, and well-being in cancer patients.

These reviews identified limitations in research design: shortened treatment procedures that differed from real time, lack of clarification about practitioner experience, poor documentation of random assignment, use of convenience samples, and underreporting of demographic as well as statistical data, which limit the possibilities for meta-analysis and generalizations to a greater population (Monroe, 2009; Peters, 1999; Winstead-Fry & Kijek, 1999).

## HEALING TOUCH RESEARCH

Review of HT research demonstrated positive outcomes suggesting reduced stress, anxiety, and pain; some improved biomarkers; accelerated healing; increased perception of well-being (Wardell & Weymouth, 2004); and improved health-related quality of life (Anderson & Taylor, 2011). Natural Standard (2012b) assigned C grades—unclear evidence—for impact on pain, anxiety, and stress.

Appealing for the development of quality research that can demonstrate both effectiveness and therapeutic potential of HT, reviewers emphasized the importance of addressing methodologic issues such as the consequences of blinding and placebo treatments, the value of both quantitative and qualitative data, and the implications of standardized vs. individualized therapy (Anderson & Taylor, 2012).

## REIKI RESEARCH

A review of Reiki research found promising outcomes for pain and possible influence on biological markers (Vitale, 2007). A systematic review of RCTs described insufficient evidence for establishing efficacy (Lee, Pittler, & Ernst, 2008). Natural Standard (2012c) assigned a C grade, suggesting some benefit for cancer quality of life, pain, and fatigue.

Employing rigorous trial designs for future efficacy studies (Lee, Pittler, & Ernst, 2008), using mixed methods data collection, balancing quantitative with qualitative studies to explain findings, considering the potential treatment activity of placebo Reiki treatments beyond placebo, and identifying specific treatment protocol used would strengthen study findings (Vitale, 2007).

## Integrative Evidence

Therapeutic Touch has the most evidence via published research since 1975, HT has some well-designed high-quality studies, and Reiki is catching up. Still, the verdict for all three appears to be "too little evidence for efficacy." Study design quality and challenges with meeting RCT standards of blinding and placebo control have contributed to problems demonstrating efficacy. Based on these outcomes, we cannot conclude that one of the three therapies is more efficacious than another. As separate entities with distinct philosophical and technical approaches, the issue of determining efficacy may not lie in differences but rather in similarities.

Jain and Mills (2010) conducted a rigorous systematic review of 66 peer-reviewed published clinical studies of biofield therapies (including TT, HT, and Reiki) with different patient populations. The quality rating score included 16 total possible points addressing methodology and design, both statistical methods and outcome methods. This included RCTs and pre- and postmeasure within-subject designs. Three of the criteria from Jadad et al. (1996)—double-blinding, randomization procedures, and description of dropouts—were applied as appropriate depending on the type of study. The review found that the quality scores were medium (m = 6.4). The studies demonstrated strong evidence (Level 1) for pain-intensity reduction in pain populations and moderate evidence (Level 2) for pain-intensity reduction in hospitalized and cancer populations.^1^ For affective behaviors, the studies demonstrated moderate evidence for lowering anxiety in hospitalized patients and moderate evidence for lessening negative behavioral symptoms associated with dementia (Jain & Mills, 2010).

Jain and Mills (2010) also observed that decreasing pain intensity demonstrated the strongest evidence for biofield therapy efficacy. They suggested the need for further research on the affective components of pain perception and quality of life as a primary measure before making inferences about the effects of biofield interventions on quality of life in pain patients. Further functional measures and biomarkers relevant to particular pain disorders would enhance our understanding of efficacy. With moderate evidence for efficacy with acute cancer pain, Jain and Mills (2010) suggested further study of long-term effects of biofield therapies on cancer pain. They also recommended physiologic studies of biofield therapies and the relaxation response.

## Studies Relevant to Oncology

A review of clinical research since 2004 addressing cancer-related symptoms and a recent in vitro study gives a picture of current research on TT, HT, and Reiki and oncology-related concerns (see Table 2).

**Table 2:**
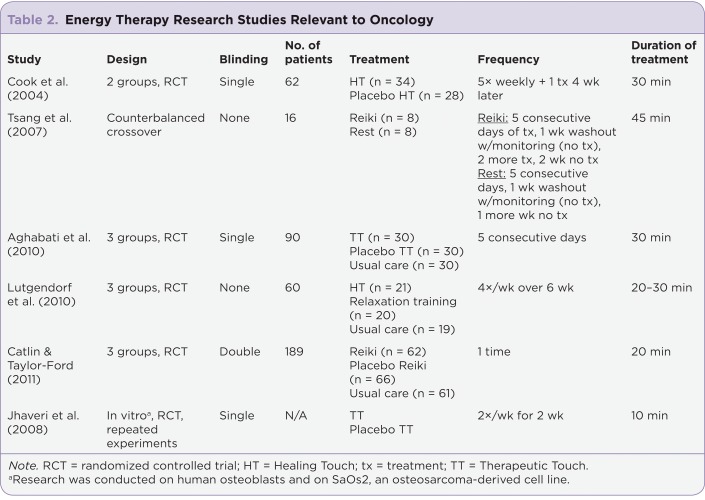
Table 2. Energy Therapy Research Studies Relevant to Oncology

## CANCER-RELATED SYMPTOMS

A study of HT and quality of life for women receiving radiation treatment for gynecologic or breast cancer, with subjects and research assistant blinded to treatment, demonstrated a significant increase mean change in health function (t = 8.13, * p* = .00) with HT treatment compared to placebo^2^ treatment (t = 1.13, not significant; overall Short Form Health Survey [SF-36] score; Cook, Guerrerio, & Slater, 2004). For both HT and placebo treatment, a 3 × 3 ft screen (like those used in surgery) was placed between head and body to shield recipients from seeing who was providing the treatments and how they were given. Healing Touch treatments were administered without physical contact. The placebo treatment mimicked HT by having the clinician walk around the recipient without placing hands in the energy field. Treatment consisted of four phases: healer preparation, energetic assessment, HT intervention, and posttreatment assessment.

A pilot study on cancer fatigue assigned participants to Reiki or resting control groups. Participants had been diagnosed with cancer in stages I to IV, had recently completed chemotherapy, and had reported during screening that they were experiencing fatigue (Tsang, Carlson, & Olson, 2007). Treatments were given by a Reiki Master with 10 years’ experience. Fatigue decreased within the Reiki treatment group over the course of all seven treatments (FACT-F, * p* = .05; effect size 0.56), and quality of life showed significant improvements (FACT-G, * p* < .005). Daily symptom monitoring before and after each treatment session demonstrated significant decreases in tiredness (* p* < .001), pain (* p* < .005), and anxiety (* p* < .01) for the Reiki treatment period only.

A RCT of female patients undergoing chemotherapy tested differences in pain (measured by a visual analog scale) and fatigue among three treatment groups (Aghabati, Mohammadi, & Pour Esmaiel, 2010): TT, placebo TT, and control (usual care). Placebo TT imitated TT movements while the naive practitioner counted backward from 100. The TT intervention, replicating TT in the natural setting, followed the standard TT process: centering, assessment, administration, reassessment, and additional treatment as needed. A repeated-measures analysis of variance revealed a significant linear decrease in pain (F = 2.01, degrees of freedom [df] = 8, * p* = .04) and fatigue (F = 3.18, df = 8, * p* = .002) compared with placebo and control over the 5 treatment days. Smaller significant differences from placebo to control were equated with placebo effect.

A prospective RCT of women receiving chemoradiation for cervical cancer compared the effect of HT, relaxation training, and usual care on cellular immunity support, mood, and quality of life; treatment-associated toxicities; and resultant treatment delay in three treatment arms: HT, relaxation training, and usual care (control). Interventions were administered immediately following radiation (Lutgendorf et al., 2010). Treatments administered by certified HT practitioners included commonly used techniques: grounding and centering, pain drain, (a technique that allows a practitioner to energetically drain off pain from an area of discomfort), chakra connection, magnetic unruffling, and mind clearance. Assessment was not mentioned as part of treatment.

The HT patients did not experience immune system depression (minimal decrease in natural killer cell cytotoxicity [NKCC]) as was experienced by the relaxation and usual care patients (sharp decline in NKCC activity; group by time interaction: * p* = .018). Two indicators of depressed mood were significantly decreased (* p* < .05) in the HT group compared to placebo treatment and control. Anxiety decreased for all groups over time. The authors suggested that with HT, "there was some effect of manipulation of hypothesized biofields, resulting in preservation of the innate immune response in HT recipients" (Lutgendorf et al., 2010, p. 9). Hart, Freel, and Lutgendorf (2011) thoroughly described their methodology in a subsequent article.

A double-blind RCT with 189 participants receiving chemotherapy compared the three treatments for outcomes of comfort and well-being (Catlin & Taylor-Ford, 2011). Both the treatment and placebo groups used a standard Reiki hand placement protocol. The placebo therapist, who visually resembled the actual therapist and specifically did not believe in Reiki, practiced mental distraction. Well-being and comfort (* p* < .05) improved for both treatment groups pre- to posttreatment compared to the usual care control, with no significant difference between Reiki and placebo Reiki. After considering the possible therapeutic energy activity of the placebo treatment, the researchers concluded that registered nurse support was the active variable.

## IN VITRO STUDY

Jhaveri, Walsh, Wang, McCarthy, and Gronowicz (2008) conducted a single-blind in vitro RCT of TT on human osteoblasts (HOBs) and on an osteosarcoma-derived cell line (SaOs2). A highly controlled and rigorous study design demonstrated a significant (* p* = .03) increase in HOB DNA synthesis compared to controls after four treatments, and at 2 weeks, increased mineralization of HOBs (* p* = .016) and decreased mineralization of SaOs2 (* p* = .0007) compared to controls. The researchers observed that TT "appears to increase human osteoblast DNA synthesis, differentiation and mineralization, and decrease differentiation and mineralization in a human osteosarcoma-derived cell line" (Jhaveri et al., p. 1541). Monzillo and Gronowicz (2011) extensively described their methodology in a subsequent article.

## Discussion

The findings from the first four studies discussed reported a positive benefit for oncology patients in the realms of quality of life and health function for women receiving radiation treatment (HT; Cook et al., 2004); fatigue and quality of life for patients who have completed chemotherapy (Reiki; Tsang et al., 2007); pain and fatigue in women receiving chemotherapy (TT; Aghabati et al., 2010); and improved mood and innate immune response preservation (HT; Lutgendorf et al., 2010). Patients treated with hands-on touch while receiving chemotherapy demonstrated improved comfort and well-being from actual as well as placebo treatments (Reiki; Catlin & Taylor-Ford, 2011). Human osteoblasts demonstrated increases in DNA synthesis and mineralization, while osteosarcoma-derived cells showed a verifiable decrease in mineralization (TT; Jhaveri et al., 2008).

These studies demonstrate a response to previous research critiques with stronger research designs, consideration of placebo, use of experienced practitioners, clearer description of interventions, and consideration of dose and timing. Cook et al. (2004) created a unique placebo treatment utilizing a surgical screen, decreasing possible energy field interaction from placebo. For placebo Reiki, Catlin and Taylor-Ford (2011) used physical touch, which confounded findings and reinforced the problem of energy field interaction with placebos. Even though the placebo practitioner did not believe in energy healing, could belief in the nurse/patient relationship confound the placebo treatment experience? Could that be successfully eliminated from an interaction that uses touch?

The crossover treatment design used by Tsang et al. (2007), allowing for natural treatment without the artificial conditions required by placebo, considered dose by giving treatments over five consecutive days. This is how Reiki treatments are traditionally given. Additionally, in traditional Reiki, family members are attuned to Reiki, so they can continue treatments without the practitioner. The design of the Aghabati et al. (2010) study included full natural TT treatment steps and, similar to the Tsang et al. (2007) study, considered daily dose over time. Although assessment was not mentioned by Lutgendorf et al. (2010), their study participants received a specific set of treatments traditionally offered by HT practitioners, with naturalistic dose frequency (4 times weekly) over time (6 weeks).

Lutgendorf et al. (2010) demonstrated directionality of healing by improved cellular immunity in the energy treatment group. Similarly, Jhaveri et al. (2008) found that healthy cells got healthier while unhealthy cancer cells actually deteriorated compared to placebo treatment. This corroborates the explanation that biofield therapies support restoration of balance by strengthening healthy cells, a mark of restoration toward wholeness.

These RCTs represent essential information for EIP. Foremost, the research literature demonstrates the safety of these therapies. Further, as study quality improves, research continues to demonstrate efficacy for symptoms commonly associated with cancer: pain, anxiety, quality of life, and function.

## Energy Therapies in Integrative Oncology Practice

The therapies discussed here demonstrate some efficacy for pain and anxiety relief. Possessing no known side effects or potential interaction with pharmaceuticals and requiring no energy expenditure on the part of the patient, biofield therapies may be more appropriate than cognitive therapies for those patients experiencing cognitive impairment due to a malignancy or its treatment (Anderson & Taylor, 2011).

Progressively, more biofield therapies are offered in the integrative cancer care setting. Pierce (2007) attributes the implementation of biolfield therapies in the clinical setting to the growing numbers of nurses who practice these therapies. Several hundred thousand practitioners have been trained in HT, TT, and Reiki since the 1970s. Although the number of nurses offering biofield therapies in oncology settings is unknown, some of them practice in established integrative medicine programs. Three examples of such programs are described in what follows.

## STANFORD MEDICAL CENTER

The Healing Partners Program at Stanford Medical Center offers HT for women with breast cancer (Turner, 2005). Healing Partners is modeled after an established program in Hawaii called "Bosom Buddies." Paired with an HT volunteer, women receive free weekly sessions for 6 months. Participants in this program report deep relaxation, stress relief, reduced physical symptoms, increased tolerance for medical procedures, more rapid recovery from surgery, better sleep, and more energy for tasks of daily living. Healing Touch volunteers describe benefit from the satisfaction they feel from having a positive impact on their partner’s quality of life and from the mutual benefit they receive through sharing the journey of healing. After recovery, recipients often choose to train in HT so they can volunteer to be Healing Partners for other women with breast cancer. 

## BRITISH COLUMBIA CANCER AGENCY

The British Columbia Cancer Agency has offered TT for more than 20 years. Stephen et al. (2007) asserted that the success of TT in this cancer research institution came from placing TT within the context of evidence-based practice, collecting satisfaction data as well as designing studies to better understand the phenomenon; developing and ensuring professional practice standards through a supervised TT volunteer program; and avoiding taking a partisan stance on TT by communicating the idea of placebo effect as a plausible explanation for its value to patients.

Therapeutic Touch was initiated at the agency in 1985, when two counselors, having received TT training from Krieger, began to teach the counselors and nursing staff, who then continued through advanced training. Patients could schedule TT sessions and TT was available to them "on call" for urgent requests. Data collected in 1995 and 1998 from this program indicated that patients primarily used TT for treatment-related anxiety. They used it for improved well-being and for coping with pain, insomnia, and needle phobia. Therapeutic Touch provided valued supportive care; patients consistently reported "feelings of relaxation and calm," which helped to relieve treatment-associated side effects. In the face of increasing patient demand, providing TT by paid counselors became prohibitive. Currently, group sessions are offered at specified times in a relaxing environment by TT volunteers. Patients continue to report increased relaxation and decreased pain and anxiety. Satisfaction data support continued availability of this therapy.

## LEONARD P. ZAKIM CENTER AT DANA-FARBER CANCER INSTITUTE

Reiki is offered at the Leonard P. Zakim Center for Integrated Therapies for the Pain and Palliative Care Program, Dana-Farber Cancer Institute (Bossi, Ott, & DeCristofaro, 2008). Patients—whether they are receiving standard or experimental cancer therapies—are referred to Reiki treatment by clinicians or by self-referral. Outpatients pay a nominal fee, although some qualify for free treatments. Treatments are offered in private clinic rooms or in treatment areas before or after a medical intervention. Pretreatment assessment and posttreatment response are documented in the medical record. Common referrals for symptom management include pain, anxiety, nausea, and sleep disturbance. No negative side effects have been reported. Patients spontaneously describe positive benefits, which include feeling peaceful and relaxed, feeling decreased anxiety for making treatment decisions, improved sleep quality, decreased pain, increased mobility with peripheral neuropathy, strengthened self-perception, increased receptivity to other complementary and alternative modalities, and increased peaceful feelings at the end of life.

## Research Directions

We must continue to conduct the basic science cell-line studies with experienced TT, HT, or Reiki practitioners; test biological markers for immunity and stress; and choose the best instruments for measuring psychosocial outcomes (anxiety, depression, fatigue, and function). When utilizing placebo, we should create interventions that are truly inert (e.g., Cook et al., 2004) and drop the monikers of "mock" and "sham" from our descriptions; they demean the science.

We know that by attempting to isolate aspects of energy field therapy phenomena, RCT design has been challenged to demonstrate efficacy. Recognizing the value and limitations of reductionist research as providing only one aspect of understanding a phenomenon, triangulation to include the patient’s experience is essential for interpreting outcomes. In addition, creating more naturalistic studies that evaluate the use of the therapy as it is normally practiced would allow for the inclusion of therapist as a variable and might also demonstrate the unique distinctions among these therapies.

## State of the Evidence

This article represents an essential starting point for gathering evidence for EIP and energy therapies in oncology by presenting one practitioner’s experience and expertise with these modalities as well as a review of pertinent current published research on the modalities. This evidence suggests benefit with no evidence suggesting harm, and it provides recommended directions for further research.

Further evidence may be gleaned from the laboratory of self by scheduling two or more sessions with a credible energy therapist. Knowledge of these energy therapies is essential for advanced oncology practitioners in the pursuit of evidence-informed practice. Evidence-informed understanding ensures that the recommendations made and the responses given to patient inquiries come from an informed place rather than from the dismissive bias that can be attendant with unfamiliarity.

The next steps require fleshing out the experience of these modalities by patients and health-care providers in the oncology care setting. Qualitative case reports, epidemiologic outcomes, and health services research will add meaning, association, and utilization data. To consider the varieties of evidence for TT, HT, and Reiki, EIP requires a qualitative examination of patient experiences with these modalities, exploration of where these modalities have been integrated into cancer care and how the practice works in the oncology setting, and discovery of the impact of implementation on provider practice and self-care.

## The Author’s Personal Experience

I have long described myself as a hybrid healer (Potter, 2003). Through TT, I learned to assess the energy field with my hands, and I learned the importance of centering, grounding, and protecting myself from imbalanced energies. Through HT, I learned about the *chakras* and their metaphorical connection to a person’s manifestation of symptoms; I learned a variety of techniques to address the myriad ways energy imbalances present. Adding Reiki to my energy therapy repertoire surprised me. Reiki took the "try" out of the experience. I no longer had to so conscientiously prepare for an energy encounter. Reiki was always present, relaxing, balancing, and clearing, both the recipient and myself, whenever I initiated a treatment. Energy work became energizing. When I treat, I use techniques from TT and HT. Mostly I allow the universal life energy, Reiki, to flow, finding its own direction and level.

While a graduate student at Yale School of Nursing, I became the lead Reiki volunteer at Yale New Haven Hospital, where I began offering Reiki on the oncology floor. The nursing staff, at first aloof and skeptical, saw the relief their patients experienced and began to ask me to treat specific patients who were experiencing discomfort. We had a similar positive response to other Reiki volunteers.

After establishing the volunteer program on the unit, I was asked to offer Reiki to the women in the gynecologic oncology clinic. Beginning with a circle of eight women who were receiving chemotherapy for varying stages of ovarian cancer, I approached each one this way: "I offer a therapy called Reiki. It might help you relax." As I moved around the room, one woman at a time, the energy in the space began to change, to grow calmer. At times, I provided comfort and calm distraction for the new patient while the nurses established her intravenous line and initiated treatment. Another time, after watching me treat several women, a woman who was in obvious distress from the pressure of ascites and her deteriorating condition asked if I would treat her. She especially complained of back pain. I treated her head to toe, returning to her mid and lower back for my final hand positions. She rested deeply. After I moved on to the next patient, the woman said to me, "I can still feel the heat of your hands, like they are still there on my back." 

To me, Reiki offers a remembered wellness, an opportunity, for a moment, to feel stilled and whole. No, it doesn’t cure—and it doesn’t claim to. Yes, it comforts. And within that comfort, it facilitates healing.

I am a nurse, scientist, and energy healer. As an advanced practice psychiatric nurse working with people experiencing cancer, I am interested in the mental/emotional well-being of my patients. As a scientist, I recognize the importance of rationally demonstrating efficacy, or the lack of it, for the therapies that we offer patients. We want to increase the possibility of healing and diminish the possibility of harm. My experience with these therapies—therapeutic touch, healing touch, and Reiki—is that they help people feel better and they offer comfort in the midst of diagnosis, treatment, and recovery. Above all, they do no harm.

## Acknowledgments

The author would like to express her humble appreciation and thanks to the patients who have participated in these studies; to all of the people who have conducted the studies, written and thought about the nature of energy therapies, and asked the questions about how we can better understand modes of action and outcomes; and to everyone who wrote integrative reviews of the research.

## References

[1] Aghabati Nahid, Mohammadi Eesa, Pour Esmaiel Zahra (2010). The effect of therapeutic touch on pain and fatigue of cancer patients undergoing chemotherapy.. *Evidence-based complementary and alternative medicine : eCAM*.

[2] Anderson Joel G, Taylor Ann Gill (2011). Effects of healing touch in clinical practice: a systematic review of randomized clinical trials.. *Journal of holistic nursing : official journal of the American Holistic Nurses’ Association*.

[3] Anderson Joel G, Taylor Ann Gill (2012). Biofield therapies and cancer pain.. *Clinical journal of oncology nursing*.

[4] Barnett L., Chambers M. (1996). *Reiki energy medicine: Bringing Healing Touch into home, hospital, and hospice.*.

[5] Bossi Larraine M, Ott Mary Jane, DeCristofaro Susan (2008). Reiki as a clinical intervention in oncology nursing practice.. *Clinical journal of oncology nursing*.

[6] Bravewell Collaborative (2011). What is integrative medicine? The Bravewell Collaborative.. http://www.bravewell.org/integrative_medicine/what_is_IM/.

[7] Catlin Anita, Taylor-Ford Rebecca L (2011). Investigation of standard care versus sham Reiki placebo versus actual Reiki therapy to enhance comfort and well-being in a chemotherapy infusion center.. *Oncology nursing forum*.

[8] Coakley Amanda Bulette, Barron Anne-Marie (2012). Energy therapies in oncology nursing.. *Seminars in oncology nursing*.

[9] Cook C., Guerrerio J. F., Slater V. E. (2004). Healing Touch and quality of life in women receiving radiation treatment for cancer: A randomized controlled trial.. *Alternative Therapies in Health & Medicine*.

[10] Fouladbakhsh J. M., Stommel  M. (2010). Gender, symptom experience, and use of complementary and alternative medicine practices among cancer survivors in the U.S. cancer population.. *Oncology Nursing Forum*.

[11] Hart Laura K, Freel Mildred I, Haylock Pam J, Lutgendorf Susan K (2011). The use of healing touch in integrative oncology.. *Clinical journal of oncology nursing*.

[12] Healing Touch International (2012). *Healing Touch International, Inc*.

[13] Healing Touch Program (2012). *Healing Touch program*.

[14] Jadad A R, Moore R A, Carroll D, Jenkinson C, Reynolds D J, Gavaghan D J, McQuay H J (1996). Assessing the quality of reports of randomized clinical trials: is blinding necessary?. *Controlled clinical trials*.

[15] Jain Shamini, Mills Paul J (2010). Biofield therapies: helpful or full of hype? A best evidence synthesis.. *International journal of behavioral medicine*.

[16] Jhaveri Ankur, Walsh Stephen J, Wang Yatzen, McCarthy MaryBeth, Gronowicz Gloria (2008). Therapeutic touch affects DNA synthesis and mineralization of human osteoblasts in culture.. *Journal of orthopaedic research : official publication of the Orthopaedic Research Society*.

[17] Jonas Wayne B (2005). Building an evidence house: challenges and solutions to research in complementary and alternative medicine.. *Forschende Komplementärmedizin und klassische Naturheilkunde = Research in complementary and natural classical medicine*.

[18] Lee M S, Pittler M H, Ernst E (2008). Effects of reiki in clinical practice: a systematic review of randomised clinical trials.. *International journal of clinical practice*.

[19] Lutgendorf Susan K, Mullen-Houser Elizabeth, Russell Daniel, Degeest Koen, Jacobson Geraldine, Hart Laura, Bender David, Anderson Barrie, Buekers Thomas E, Goodheart Michael J, Antoni Michael H, Sood Anil K, Lubaroff David M (2010). Preservation of immune function in cervical cancer patients during chemoradiation using a novel integrative approach.. *Brain, behavior, and immunity*.

[20] Monroe Carolyn Magdalen (2009). The effects of therapeutic touch on pain.. *Journal of holistic nursing : official journal of the American Holistic Nurses’ Association*.

[21] Monzillo E., Gronowicz G. (2011). New insights on Therapeutic Touch: A discussion of experimental methodology and design that resulted in significant effects on normal human cells and osteosarcoma. **.

[22] National Center for Complementary Alternative Medicine (2000). Five-year strategic plan 2001-2005.

[23] (2012a). Therapeutic Touch.. *Natural Standard*.

[24] (2012b). Healing Touch. *Natural Standard*.

[25] (2012c). Reiki. *Natural Standard*.

[26] Nevo I., Slonim-Nevo V. (2011). The myth of evidence-based practice: Towards evidence-informed practice.. *British Journal of Social Work*.

[27] (2012a). EIP defined. Evidence in Action, 5(Spring). *Northwestern Health Sciences University.*.

[28] (2012b). Research education. *Northwestern Health Sciences University*.

[29] Peters R M (1999). The effectiveness of therapeutic touch: a meta-analytic review.. *Nursing science quarterly*.

[30] Pierce Beverly (2007). The use of biofield therapies in cancer care.. *Clinical journal of oncology nursing*.

[31] Potter P. (2003). What are the distinctions between Reiki and Therapeutic Touch?. *Clinical Journal of Oncology Nursing*.

[32] Rand W. L. (1991). *Reiki: The Healing Touch*.

[33] Sagar Stephen M (2006). Integrative oncology in North America.. *Journal of the Society for Integrative Oncology*.

[34] Stephen Joanne E, Mackenzie Gina, Sample Sarah, Macdonald Jennifer (2007). Twenty years of therapeutic touch in a Canadian cancer agency: lessons learned from a case study of integrative oncology practice.. *Supportive care in cancer : official journal of the Multinational Association of Supportive Care in Cancer*.

[35] Straneva J A (2000). Therapeutic touch coming of age.. *Holistic nursing practice*.

[36] Therapeutic Touch International Association (2012). *Therapeutic Touch International Association.*.

[37] Tsang Kathy L, Carlson Linda E, Olson Karin (2007). Pilot crossover trial of Reiki versus rest for treating cancer-related fatigue.. *Integrative cancer therapies*.

[38] Turner K. (2005). Healing Touch for breast cancer patients.. *Stanford Nurse*.

[39] Vitale A. (2007). An integrative review of Reiki touch therapy research.. *Holistic Nursing Practice*.

[40] Wardell Diane Wind, Weymouth Kathryn F (2004). Review of studies of healing touch.. *Journal of nursing scholarship : an official publication of Sigma Theta Tau International Honor Society of Nursing / Sigma Theta Tau*.

[41] Winstead-Fry P, Kijek J (1999). An integrative review and meta-analysis of therapeutic touch research.. *Alternative therapies in health and medicine*.

